# Salivary physicochemical characteristics and antimicrobial human peptide among Indian children with dental caries

**DOI:** 10.6026/97320630019428

**Published:** 2023-04-30

**Authors:** Vatchala Rani RM, Neha Singh, Swati Murmu, Abhishek, Sakshi Raina, Shalini Singh

**Affiliations:** 1 Department of Oral Pathology and Microbiology Faculty of Dentistry, Jamia Millia Islamia, New Delhi, India; 2 Tutor, Department of Periodontology, Dental College, Rims, Ranchi, Jharkhand, India; 3 Post Graduate Student, Department of Paediatric and Preventive Dentistry, Hazaribag College of Dental Sciences and Hospital, Hazaribagh, Jharkhand, India; 4 Private Practioner, Oral and Maxillofacial Surgeon, Oro Care Facial Trauma Centre, Patna, Bihar, India; 5 Department of Orthodontics, Dental Officer, Echs Polyclinic (Ministry of Defence) Samastipur, Bihar, India; 6 Senior Resident, Department of Pedodontist, Safdarjung Hospital, New Delhi, India

**Keywords:** Saliva, caries activity, physiochemical properties, defensive peptides

## Abstract

Salivary innate defenses encompass mechanical cleaning, calcium phosphate salts and fluoride ion reduction of enamel dissolution rate, buffering capacity and neutralizing capacity, and antibacterial properties employing antimicrobial substances like antimicrobial peptides,agglutins,lactoferrin, lactoperoxidase, lysozyme and immuno globulins. Antimicrobial peptides play a key role in the initial defensive responses that make up innate immunity. The aim of this study was to assess the relationship between salivary physicochemical characteristics like buffering capacity, pH, flow rate, and concentrations antimicrobial human peptide like HBD-3, HNP1-3 and LL-37 and caries activity in young children. Before to collecting the samples, informed permission papers were gathered and completed by the children's legal guardians or parents. There was significant correlation showing reduced caries activity on increased concentrations of HNP1-3. When there was analysis of correlation of CAS with LL-37 concentrations then the p value vas 0.002 showing the correlation was significant. There is significant relationship between salivary physicochemical characteristics like buffering capacity, pH, flow rate, and concentrations antimicrobial human peptide like HBD-3, HNP1-3 and LL-37 and caries activity in young children.

##  Background:

Dental caries is among the most challenging issues to handle throughout a child's first few years of life. It has been demonstrated that a number of factors are helpful in reducing children's dental caries risks both early on and later on.. Salivary ingredients among them are recognized to play a significant role in mediating and eventually lowering the hazards associated with caries start and development. Salivary innate defenses encompass mechanical cleaning, calcium phosphate salts and fluoride ion reduction of enamel dissolution rate, buffering capacity and neutralizing capacity, and antibacterial properties employing antimicrobial substances like antimicrobial peptides, agglutins, lactoferrin, lactoperoxidase, lysozyme and immuno globulins. [1,2]Antimicrobial peptides play a key role in the initial defensive responses that make up innate immunity. [3, 4] Based on the type of amino acids and cross sectional three-dimensional geometry, there are three main categories of antibacterial biopeptides: those with unusually high percentages of specific amino acids, like histatins; those with three disulfide covalent bonds, like the - defensins and those with -helical peptides that do not contain cysteine, like cathelicidins.

Recent research suggests that whereas histatins are largely antifungal compounds, defensins biomolecules and cathelicidins biomolecules have an antibacterial property in mouth. [5, 6 7] Human beta-defensins biomolecules considered as HBDs are frequently found in epithelium of gingival sulcus and oral mucosa.[8, 9, 10] Ducts of salivary gland , major salivary glands, and their contents all contain HBD-1 biomolecule and HBD-2 biomolecule. [11,12] The HBD-3 biomolecule, a member of the innate immune peptide group, is a relatively recent discovery in research and has recently drawn more attention. This compound is generated either intrinsically or by activation. This peptide biomolecule exhibits antimicrobial potential towards Gram-negative bacteria and Gram-positive bacteria as well as serving as chemo-attractant. [13]The fluid from gingival crevices contains alpha-defensins, also known as Human Neutrophil Peptides (HNP13), which are found in neutrophil lymphocytes and are involved in non - oxidative bacterial mortality. [14,15] Furthermore, certain salivary proteins make saliva viscous as the pH drops, shielding the teeth from acidic loads by creating a physical shield. [16,17] Therefore, it is of interest to assess the relationship between salivary physicochemical characteristics like buffering capacity, pH, flow rate, and concentrations antimicrobial human peptide like HBD-3, HNP1-3 and LL-37 and caries activity in young children.

## Materials and Methods:

Before to collecting the samples, informed permission papers were gathered and completed by the children's legal guardians or parents. There were 82 patients, ranging in age from 3 to 12, total. Following were the criteria for inclusion: Young kids shouldn't be undergoing orthodontic therapy 1) be medically fit and active; (2) have perfectly natural tooth anatomy; (3) have primary dentition or mixed dentition; (4) be free of diseases that influence salivation, such as allergies, medications affecting salivary flow, radio therapy, surgery of salivary glands and diabetes (5) are in good general health. Kids who did not cooperate sufficiently throughout the examination or saliva assessment as well as any kids with associated systemic disorders were excluded. It was noted how often people consumed sugary snacks or drinks as well as how often they brushed their teeth. Visual/tactile criteria were used to examine the complete oral cavity for dental caries, and when necessary, routine radiographic views were included. Each study participants DMFS ratings and dmfs ratings were noted, and the total of each patient's decaying tooth surface, absent tooth surface, and filled tooth surface (DMFS + dmfs) was used to calculate the caries activity score (CAS). Saliva was simply gathered into the designated laboratory bottle to collect un aroused saliva.

All samples were gathered between 9:00 a.m. and 1:00 p.m. During at least one hour beforehand of the examination, kids were instructed to refrain from eating, drinking, or brushing their teeth; nevertheless, they were instructed to wash their mouths with still water prior to collection of saliva. Under ambient daylight, sampling was done with the kid seated in an upright, comfortable position. They were instructed to spit into a fresh sealed container, and after five minutes, the accumulated saliva was recorded under their encoding mark. Up to 2 ml of secretions were collected, the spitting process was continued. By dividing the quantity of saliva gathered by the time it took to acquire it (five minutes), the salivary flow velocity was computed. According to the manufacturer's recommendations, the pH of saliva and buffering capacity of saliva were measured utilising GC Saliva Check marketed by Tokyo, Japan. Five minutes were spent centrifuging saliva specimens at 10,000 g. For further examination, cleared; unfractionated specimens of saliva were refrigerated at 80°C. By employing an enzyme-linked immunoassay technique considered as ELISA using a quantifiable sandwich ELISA approach with an analytical kit available in the market tailored for these peptides, the quantities of HBD-3 biomolecule, HNP1-3 biomolecule, and LL-37 biomolecule and were assessed.

## Statistical analysis:

Centering values and variability values have been reported using descriptive statistics, and the averages of the gender groups were compared using an independent sample t-test statistical test and the Mann-Whitney U test. The Pearson correlation tests, Spearman's tests and linear regression, were used to determine the association among CAS values and salivary parameters as well as the association among salivary parameters and age. Using a t-test with an independent sample, the averages of CAS for both low and high-value categories of salivary items were compared. The relationship among CAS values and salivary indicators on a given scale was assessed using the chi-square test. With SPSS version 21, all statistical tests were carried out (IBM, USA). All statistical tests had a specified level of significance at P value less than 0.05.

## Results:

The mean values of CAS (DMFS + dmfs) were 23.31 (24.21 in boys and 22.52 in girl). The mean values of Flow rate was 2.57 ml/min (2.74 in boys and 2.48 in girls). The mean values of pH were 7.41 (7.46 in boys and 7.38 in girl). The mean values of buffering capacity were 9.76 (10.54 in boys and 8.61 in girl). The mean values of HBD3 were 593.75 ng/ml (494.73 in boys and 617.81 in girl). The mean values of HNP1-3 were 38.26ng/ml (44.32 in boys and 35.12 in girl). The mean values of LL-37 were 19.32 ng/ml (16.52 in boys and 19.77 in girl). The difference among boys and girls was non-significant statistically. (Table 1)

When there was analysis of correlation between age and CAS then the p value was 0.001 showing significant correlation. When there was analysis of correlation between age and salivary flow rate then the p value was 0.003 showing significant correlation. When there was analysis of correlation between age and pH then the p value was 0.005 showing significant correlation. When there was analysis of correlation between age and buffering capacity then the p value was 0.009 showing significant correlation. When there was analysis of correlation between age and concentration of HBD3 then the p value was 0.008 showing significant correlation. When there was analysis of correlation between age and concentration of HNP1-3 then the p value was 0.004 showing significant correlation. When there was analysis of correlation between age and concentration of LL-37 then the p value was 0.001 showing significant correlation. (Table 2)

When there was analysis of correlation of CAS with salivary flow rate then the p value vas 0.006 showing the correlation was significant. When there was analysis of correlation of CAS with pH then the p value vas 0.008 showing the correlation was significant. When there was analysis of correlation of CAS with buffer capacity then the p value vas 0.002 showing the correlation was significant. When there was analysis of correlation of CAS with HBD 3 concentrations then the p value vas 0.008 showing the correlation was significant. When there was analysis of correlation of CAS with HNP1-3 concentrations then the p value vas 0.001 showing the correlation was significant. There was significant correlation showing reduced caries activity on increased concentrations of HNP1-3. When there was analysis of correlation of CAS with LL-37 concentrations then the p value vas 0.262 showing the correlation was significant (Table 3).

## Discussion:

Salivary innate defenses include mechanical cleansing, calcium phosphate salts, fluoride ions, buffering and neutralizing capacity, and antibacterial characteristics using antimicrobial peptides, agglutins, lactoferrin, lactoperoxidase, lysozyme, and immuno globulins, and mechanical cleaning. [18] In the early defensive reactions that make up innate immunity, antimicrobial peptides are essential. Based on the type of amino acids and cross sectional three-dimensional geometry, there are three main categories of antibacterial biopeptides: those with unusually high percentages of specific amino acids, like histatins; those with three disulfide covalent bonds, like the defensins; and those with -helical peptides that lack cysteine, like cathelicidins. [19,20] According to recent study, histatins are mostly antifungal substances, whereas defensin and cathelicidin macromolecules have an antibacterial property in the mouth. The purpose of this study was to examine the association between caries activity in young children and salivary physicochemical features such buffering capacity, pH, flow rate, and concentrations of antimicrobial human peptides like HBD-3, HNP1-3, and LL-37.

In this study the mean values of CAS (DMFS + dmfs) were 23.31 (24.21 in boys and 22.52 in girl). The mean values of Flow rate was 2.57 ml/min (2.74 in boys and 2.48 in girls). The mean values of pH were 7.41 (7.46 in boys and 7.38 in girl). The mean values of buffering capacity were 9.76 (10.54 in boys and 8.61 in girl). The mean values of HBD3 was 593.75 ng/ml (494.73 in boys and 617.81 in girl).. The mean values of HNP1-3 were 38.26ng/ml (44.32 in boys and 35.12 in girl). The mean values of LL-37 were 19.32 ng/ml (16.52 in boys and 19.77 in girl). The difference among boys and girls was insignificant statistically showing there is no vital difference between male and female study participants.

Salivary pH was substantially higher in the caries-free category as opposed to the low cavities and nursing caries categories, according to Animireddy *et al*. [1]. In the low cavities and nursing caries categories, there was, unfortunately, no difference that was statistically significant. In children aged 7 to 14, Prabhakar *et al*. [21] showed that there was no significant difference in pH, buffering capacity, or salivary flow rate between the caries-free and caries-active groups. Thaweboon *et al*. [22] showed that there was no statistically significant difference in pH or salivary flow rate between 5-10-year-olds with widespread caries and those without it.According to Tayab *et al*. [23], the caries-active group's salivary pH, salivary buffering capacity, and flow rate of saliva were all considerably lower than those of the caries-free category.

Saliva polypeptides can act as buffers as the pH varies according to the respective net charge. Protons can either be accepted by polypeptides below their ionic strength or released just above it. Even though many salivary polypeptides are efficient buffers and have isoelectric points between pH 5 and pH 9, particularly at acidic pH values. Also, as the pH decreases, specific salivary proteins make saliva viscous, providing a physical barrier that protects the teeth from acidic loads. [22, 23, 24, 25].

The total of each patient's decayed tooth surface, absent tooth surface, and filled tooth surface (DMFS + dmfs) was used to construct the caries activity score in this investigation. Each study participant's DMFS and dmfs ratings were noted (CAS). To obtain unaroused saliva, saliva was simply collected into the proper laboratory bottle. Between 9:00 a.m. and 1:00 p.m., all samples were collected. Children were instructed to avoid from eating, drinking, or brushing their teeth for at least an hour before to the test; however, they were instructed to rinse their mouths with still water prior to the collection of saliva. The child was seated straight and at ease while sampling was being done in ambient light. After five minutes, the collected saliva was logged under each participant's encoding mark, and they were told to spit into a brand-new, sealed container. Spitting persisted until up to 2 ml of secretions were collected.

The salivary flow velocity was calculated by dividing the amount of saliva collected by the amount of time it took to collect it (five minutes). The pH of saliva and its buffering ability were tested using the Tokyo, Japan-made GC Saliva Check, as per the manufacturer's instructions. Saliva samples were centrifuged at 10,000 g for five minutes. Cleared, unfractionated saliva samples were stored in a refrigerator at 80°C for later analysis. The amounts of the biomolecules HBD-3, HNP1-3, and LL-37 were measured using an enzyme-linked immunoassay technique known as ELISA using a quantitative sandwich ELISA approach using an analytical kit commercially available for these peptides. The gingival sulcus epithelium and oral mucosa typically contain human beta-defensin biomolecules, also known as HBDs. [20,26].

HBD-1 and HBD-2 biomolecules are present in the major salivary glands, their contents, and their ducts. The HBD-3 biomolecule, a member of the innate immune peptide group, is a relatively recent discovery in research and has recently attracted more attention. This substance can be produced natively or by activation. This peptide biomolecule has chemo attractive and antibacterial properties towards both Gram-positive and Gram-negative bacteria. [23] Alpha-defensins, also known as Human Neutrophil Peptides (HNP13), which are present in neutrophil cells and are implicated in non-oxidative bacterial death, are present in the fluid from gingival fissures. [24, 25] Neutrophils, inflammatory epithelium, and saliva all contain the human cathelicidin bio-peptide LL37.

In this study when there was analysis of correlation between age and CAS then the p value was 0.001 showing significant correlation. When there was analysis of correlation between age and salivary flow rate then the p value was 0.003 showing significant correlation. When there was analysis of correlation between age and pH then the p value was 0.005 showing significant correlation. When there was analysis of correlation between age and buffering capacity then the p value was 0.009 showing significant correlation. When there was analysis of correlation between age and concentration of HBD3 then the p value was 0.008 showing significant correlation. When there was analysis of correlation between age and concentration of HNP1-3 then the p value was 0.004 showing significant correlation. When there was analysis of correlation between age and concentration of LL-37 then the p value was 0.001 showing significant correlation.

In current research when there was analysis of correlation of CAS with salivary flow rate then the p value vas 0.006 showing the correlation was significant. When there was analysis of correlation of CAS with pH then the p value vas 0.008 showing the correlation was significant. When there was analysis of correlation of CAS with buffer capacity then the p value vas 0.002 showing the correlation was significant. When there was analysis of correlation of CAS with HBD 3 concentrations then the p value vas 0.008 showing the correlation was significant. When there was analysis of correlation of CAS with HNP1-3 concentrations then the p value vas 0.001 showing the correlation was significant. There was significant correlation showing reduced caries activity on increased concentrations of HNP1-3. When there was analysis of correlation of CAS with LL-37 concentrations then the p value vas 0.262 showing the correlation was significant

According to Kaur *et al*. [24], 90 percent of 4-6 year olds with no caries and 33 percent of those with active caries have regular salivary flow rates. It was found that 100 percent of children with no caries and 30 percent of kids with active caries had adequate pH, with the variations being statistically meaningful. These values were reported to be 120-12,000, 0-6210, and 60-10,500 by Tao *et al*. [20]. According to Phattarataratip *et al*. [25], the ranges for concentrations of LL-37 , concentrations of HBD-3, and concentrations of HNP1-3 were 3.93-71.02 ng/ml, 0.15-11.56 ng/ml, and 548.63-5231.06 ng/ml, respectively. According to Davidopolou *et al*. [26], the concentration of LL-37 ranged from 0.22 to 275 ng/ml.Tao *et al*. [20] also documented on the role of HNP1-3 in caries vulnerability and found that HNP1-3 was considerably greater in kids who had no symptoms of cavities compared with those who did.

## Conclusion:

There is significant relationship between salivary physicochemical characteristics like buffering capacity, pH, flow rate, and concentrations antimicrobial human peptide like HBD-3, HNP1-3 and LL-37 and caries activity in young children.

## Figures and Tables

**Table 1:** Values of different physiochemical properties of saliva, defensins peptides and CAS in boys and girls

**Mean values**	**CAS (DMFS + dmfs)**	**Flow rate (ml/min)**	**pH**	**Buffering capacity**	**HBD3(ng/ml)**	**HNP1‑3(ng/ml)**	**LL‑37(ng/ml)**
All	23.31	2.57	7.41	9.76	593.75	38.26	19.32
Boys	24.21	2.74	7.46	10.54	494.73	44.32	16.52
Girl	22.52	2.48	7.38	8.61	617.81	35.12	19.77
P value	0.831	0.647	0.657	0.095	0.652	0.868	0.588

**Table 2:** Analysis of correlation of age with caries activity score and other physiochemical and peptides concentration

**Age**	**Correlation coefficient**	**P value**
CAS	−0.158	0.001
Flow rate	0.097	0.003
pH	-0.077	0.005
Buffering capacity	0.229	0.009
HBD3	-0.074	0.008
HNP1-3	-0.087	0.004
LL-37	0.388	0.001

**Table 3:** Analysis of correlation of CAS with physiochemical properties and peptides concentrations in saliva

**CAS**	**Correlation coefficient**	**P value**
Flow rate	0.068	0.006
pH	0.074	0.008
Buffering capacity	-0.025	0.002
HBD3	-0.010	0.008
HNP1-3	-0.207	0.001
LL-37	0.339	0.002
